# Hazardous gases (CO, NO_x_, CH_4_ and C_3_H_8_) released from CO_2_ fertilizer unit lead to oxidative damage and degrades photosynthesis in strawberry plants

**DOI:** 10.1038/s41598-018-30838-3

**Published:** 2018-08-16

**Authors:** Sowbiya Muneer, Jeong Hyun Lee

**Affiliations:** 1 Department of Horticulture, College of Agricultural life sciences, Chonnam National University, 300 Young Bong-Dong Buk-Gu, Gwangju, 500-757 Korea; 2 Centre for Agricultural Innovations and Adavnced Learning [VAIAL], Vellore Institute of Technology, Tamil Nadu, Vellore, 632014 India; 3 School of Bioscience and Biotechnology, Vellore Institute of Technology, Tamil-Nadu, Vellore, 632014 India

## Abstract

CO_2_ boilers/direct heating systems used in greenhouses often lead to incomplete combustion, which results in the formation of hazardous gases, such as carbon monoxide (CO), nitroxide (NO_X_) and other hydrocarbons. In this study, strawberry plants that were grown on rockwool cubes were transferred to airtight bottles and treated with CO, NO_X_, CH_4_ and C_3_H_8_ gases for 1–48 hours. Oxidative damage due to hazardous gases was observed, as indicated by H_2_O_2_ and ${{\bf{O}}}_{{\bf{2}}}^{{\boldsymbol{-}}1}$ determination. Photosynthetic pigments were reduced, and stomatal guard cells were damaged and remained closed compared to the control. The activity of other photosynthetic parameters was negatively related to hazardous gases. Reduction in the expression of multiprotein complexes was highly observed under hazardous gas treatments. This study highlighted that hazardous gases (CO, NO_X_, CH_4_ and C_3_H_8_) emitted due to incomplete combustion of CO_2_ fertilization units/or direct heating systems resulted in the formation of ROS in shoots and limited photosynthetic metabolism. We predicted that major steps must be incorporated to improve the efficiency of CO_2_ boiler/heating systems to decrease the emission of these hazardous gases and other hydrocarbons and to reduce the observed risks that are key to the reduction of crops.

## Introduction

The use of a greenhouse heater for carbon fertilization from combusted gas of a direct heating system or hot water boiler system is increased daily for higher yields of horticultural crops. The increase in the yield of CO_2_ supply from the heating system results in a higher concentration of CO_2_, thus increasing crop yield^1^. However, these systems used for CO_2_ supply often recapture low combustion, which results in the formation of potentially harmful gases, such as CO (carbon monoxide), NO_X_ (nitrogen oxide)^2^ and other hydrocarbons, which could be CH_4_ (methane) and/or C_3_H_8_ (propane)^3^. It has been reported that up to 12 ppm of these hazardous gases are not potential threats to crops, whereas if the concentration exceeds 20 ppm, they can greatly affect the crop yield^4,5^. The use of CO_2_ fertilization from a direct heating system or combusted gas from a hot water boiler system in greenhouse horticultural crops is still under process, although few reports have reported that increased levels of CO_2_ fertilization are harmful to plant productivity^4^. Although several steps have been incorporated in certain countries to develop an efficient carbon fertilization unit or heating system to reduce the emission of these hazardous gases, the use of CO_2_ from direct heating systems in Korea is still under process, which has greatly reduced the yield of crops in the past few years. Until now, it has not been documented how these hazardous gases affect photosynthetic metabolism and bio–macromolecules at the protein level and what steps should be taken to reduce the emission of these hazardous gases in several developing/developed countries where efficient CO_2_ fertilization units have not yet been incorporated. Here, we present a novel study on the production of reactive oxygen species (ROS), photosynthetic metabolism and thylakoid multiprotein complexes in strawberry plants, one of the highest crops grown throughout the year in Korea in greenhouses, as affected by hazardous gases, such as carbon monoxide (CO) and nitroxide (NO_X_), and other hydrocarbons, such as CH_4_ and C_3_H_8_.

As described above, CO_2_ fertilization from a direct heating system or hot water boiler system due to incomplete combustion results in the emission of hazardous gases, such as carbon monoxide (CO) and NO_x_. CO is a fungistatic gas that suppresses fungal growth; its effect is pathogen dependent and is greatly enhanced when CO is combined with O_2_ in the atmosphere. Since CO is oxidized to CO_2_ and is incorporated into organic acids and other products, it can directly affect the physiology of plants^6^. NO_x_ is a free radical gas that transfers electrons across biological membranes^7^. The biological function of NO_x_ is therefore the generation of reactive oxygen species (ROS)^7^. The physiological generation of ROS can occur as a byproduct of other biological reactions. ROS generation as a byproduct occurs with mitochondria, peroxisomes and other cellular elements^8^. In addition to CO and NO_x_, the incomplete combustion of the CO_2_ supply system used in greenhouses leads to the emission of hydrocarbons; however, these hydrocarbons are still unknown. The assumption is that these hydrocarbon emissions could be CH_4_ and C_3_H_8_ because CH_4_ has been observed as a freely available gas in greenhouses and has become a serious threat to crop production and to the outside environment. It is important to observe the effect of CH_4_ because of its potential harm to global warming. There is much less CH_4_ in the atmosphere than CO_2_, with approximately 1800 parts per billion (ppb) compared with an estimated 390 parts per million of CO_2_; conversely, it has been estimated that 95% of plant growth reduction is due to CH_4_ compared to other greenhouse gases. C_3_H_8_ is relatively highly contributed by industries for fuel consumption. C_3_H_8_ is not considered a greenhouse gas, so its effect was considered very low in previous studies. Conversely, in 2008, the United States of Environmental Protection Agency reported that high amounts of C_3_H_8_ used as fuel could be a factor in CO_2_ emissions along with other fossil fuels, which could affect the growth and development of plants. This led to the idea that C_3_H_8_ might be the other hydrocarbon emitted from the incomplete combustion of the direct heating systems used in greenhouses.

These hazardous gases lead to the development of reactive oxygen species (ROS), such as hydrogen peroxide (H_2_O_2_) and hydroxyl radical (OH.), as previously observed in our studies^4^. The elevation of these ROS can affect physiological metabolism, causing oxidative damage to nucleic acids, proteins and lipids^9–11^. These hazardous gases can also influence the content of secondary metabolites in plants, which could therefore affect aquatic and terrestrial ecosystems^12^. These hazardous gases affect photosynthesis and can affect multiprotein complex proteins in thylakoids carrying electrons and lipids^11^. It is well known that thylakoid membranes contain the multiprotein complex photosystems I and II, which include the reaction centers responsible for converting light energy to chemical energy, as well as the cytochrome *b6/f* complex and ATPase^13^.

Previously, we observed the reduction of photosynthesis in response to hazardous gas stresses in a dose-dependent manner and detected that strawberry plants can act as a hyper–accumulator due to increased levels of antioxidant enzymes to protect against environmental pollution, but the defense ability did not adequately reduce the oxidative damage under long-term exposure to hazardous gas stress (CO, NO_x_ and SO_2_) and effectively reduced plant development^11^. Since strawberry is a staple fruit for many countries and is grown in greenhouses throughout the year, a CO_2_ supply system from a direct heater is used to obtain a higher fruit yield. The present study suggested that the high concentration of these hazardous gases crucially produces reactive oxygen species (ROS) and drastically limits the photosynthetic activity. Therefore, we conclude that major steps should be incorporated to improve the efficiency of CO_2_ boilers/heating systems to reduce the emission of hazardous gases and other hydrocarbons, which are key in the reduction of greenhouse-grown crops. In addition, we conclude that efficient and highly advanced CO_2_ boilers/heating systems should be installed in greenhouses to elevate photosynthesis in order to improve crop productivity.

## Material and Methods

### Plant material and treatments

Young strawberry (*Fragaria* × *anansa*) plants were propagated on rockwool cube (10 × 10 × 6.5 cm) from the runner at 25 °C under fluorescent light (300 μmol m^−2^ s^−1^ of photosynthetic photon flux density) for initiating root and leaves for three weeks (diagrammatic representation in Supplementary Fig. S1) as described in our previous work^11^. The rooted strawberry plants were grown on the rockwool cubes with hydroponic nutrient solution containing (mM per litter for the macro elements): N 9.5, P 9.5, K 5.5, Ca 5.0, S 1.5, P 1.5 Mg 1.5 and (µM per litter for the micro elements): Fe 33, Mn 22, B 10, Zn 5, Cu 1.0, Mo 0.5. The nutrient solution supplied was regularly with electrical conductivity (EC) 1.0 dS m^−1^ and pH was maintained to 6.5–7.0. The plants were grown under fluorescent lights with a light intensity of 150 µmole m^−2^ s^−1^ with 16 h photoperiod and 8 h dark conditions. Six–week–old plants grown on rock–wool were transferred to air tight glass bottle (3 litres) and were supplied to 267 ppm of carbon monoxide (CO), 50 ppm of nitroxide (NO_x_), 167 ppm of CH_4_, and 67 ppm of C_3_H_8_. The concentration of these hazardous gases are not exactly same seen in the greenhouses what we used, it will vary according the environmental conditions provided or present. The concentration of these gases were selected based on our preliminary experiments^11^ where we used thsese hazardous gases in dose dependent manner^11^. The concentration of treatments were selected based on the records of strawberry growers in Korea. Therefore, different concentration of these hazardous gases: low or high in other countries can be have different effects. The strawberry plants exposed to CO, NO_x_, CH_4_ and C_3_H_8_ were harvested from 1–48 hours of treatment, leaves were separated by the order of ontogenic appearance and young leaves were considered for the study. Plant samples were immediately frozen in liquid N_2_ and stored in deep–freezer (−80 °C) for further analysis. For determination of physiological measurements such as net–photosynthesis, and F*v*/F*m* unharvested plants were used.

### Determination of H_2_O_2_ and O_2_^−1^

H_2_O_2_ and O_2_^−1^ levels were quantified according to Muneer *et al*.^11^ and Lin and Kao^14^. H_2_O_2_ concentration was measured calorimetrically using titanium sulfate and was expressed as nmol g^−1^ tissue fresh weight. H_2_O_2_ concentration was calculated using the extinction coefficient 0.28 mM^−1^ cm^−1^ and was expressed as n mole g^−1^ tissue fresh weight. O_2_^−1^ determination was measured calorimetrically using alpha aminobenzene sulphonic acid and alpha naphthylamine solution and was expressed as nmol g^−1^ tissue fresh weight. For O_2_^−1^ determination leaves of strawberry were extracted in phosphate buffer with pH 7.5, centrifuged for 5 min at 10,000 g. The resultant supernatant was then collected and 1 ml of 10 mM NH_2_OH-HCl and phosphate buffer (pH 7.8) were added. The solution was incubated at 25 °C for 1 h, incubated samples were added with 0.5 ml of 17 mM alpha aminobenzene sulphonic acid and 0.5 ml of 7 mM alpha naphthylamine solution. The mixture was again incubated for 20 min at 25 °C and absorbance was recorded at 530 nm

### Photosynthetic pigments

The content of chlorophyll and carotenoid was estimated by the method of Hiscox and Israclstam^15^ as described in our previous studies^11^.

### Measurement of photosynthetic activity, Fv/Fm ratio and Stomatal observation

Photosynthesis rate, were measured using a portable photosynthesis measurement system (LI-6400XT. LI-COR, Inc., Lincoln, NE) and F*v*/F*m* ratio were measured by modulated fluormetre (OS1–FL, Optic Science) after 30 min dark adaptataion. One of the fully expanded leaves from per plant was measured for 1, 3, 9, 24 and 48 hours after different hazardous gas (CO, NO_x_, CH_4_ and C_3_H_8_) treatments.

For stomatal observation, thin layer of leaf tissues was carefully cut and were laid on a glass slide, covered with a cover slip by adding few drops of water and were observed under light microscope under 40X magnification.

### RuBisCO activity and sucrose synthase

RuBisCO (Ribulose-1,5-bisphosphate carboxylase/oxygenase) activity in leaves was estimated by the method given by Parry *et al*.^16^. One g of leaf sample was extracted in extraction buffer containing 50 mm Bicine (pH 8.0), 20 mM KCl, 5 mM DTT, 0.1 mM EDTA, 2% PVP and 0.1 mM PMSF and 5 µM luepeptin and was centrifuged at 5 000 g for 5 min. Assays of initial activity were performed at 30 °C, with 100 mL of supernatant added to 400 mL of assay buffer (166 mM Bicine-KOH, pH 8.0, 10 mM MgCl_2_, 5 mM DTT, and 25 mM NaHCO_3_). The reaction was initiated with the addition of RuBP to a final concentration of 0.5 mM and terminated after 1 min with 200 mL of 5 N HCl. RuBisCO activity was measured following the oxidation of NADH at 340 nm.

Sucrose synthase in leaves was estimated spectrophotometricaly by the method given by Xu *et al*.^17^. Sucrose synthase was assayed in a 1 ml reaction mixture containing 100 mM MES (pH 6.5), 3 mM Mg–acetate, 0.5 mM EDTA, 5 mM 2-mercaptoethanol, 0.02 mM glucose 1,6-diP, 0.5 mM NAD, 1 mM UDP, 1 mM PPi, 50 mM sucrose (for crude enzyme), or 200 mM sucrose (for purified enzyme), 1 unit phosphoglucomutase, 2 units glucose-6-P dehydrogenase (from Leuconostoc), and 1 unit UDP–glucose pyrophosphorylase was added. Sucrose synthase activity was measured following the oxidation of pyrophosphates (PPI) at 340 nm.

### Multiprotein complex proteins (MCPs) (Thylakoid proteins)

BN-PAGE of integral thylakoid proteins was performed according to our previous studies Muneer *et al*.^4^. The leaves from strawberry treated plants were collected in a liquid nitrogen and immediately grinded to fine powder in a pestle and mortar. Aprroximately five to ten gram of powdered samples were suspended in ice cold buffer containing 330 mM sorbitol/50 mM HEPES/5 mM MgCl2/2 mM EDTA/2 mM NaF with pH adjusted to 7.8. The resulting suspension was filtered through miracloth (calbiochem, San Diego, CA, USA). The filtered solution were centrifuged at 4,500 rpm for 10 min at 4 degree and resulting pellet were resuspended in same buffer, followed by centrifugation as same in first step. The pellet obtained were resuspended in second buffer conataing 20 mM tricine, 70 mM sucrose, and 5 mM MgCl_2_ (pH 7.8) and centrifuged at 4,500 rpm for 10 min at 4 degree. The resulting pellet were then washed with washing buffer of 330 mM sorbitol, 50 mM BisTris-HCl, pH 7.0, and 0.1 mg ml^−1^ pefabloc. After washing steps, the purified protein pellet were suspended in 2% w/v n-dodecyl-ß-D-maltoside (Sigma, St. Louis, MO, USA) for solubiliztion. The insoluble material were removed by centrifugation at 13,000 rpm. The resulting protein mixture were mixed with 0.1% loading dye (5% CBB-G250, 100 mM BisTris-HCl, pH 7.0, 30% w/v sucrose and 500 mM ɛ-amino-n-caproic acid) and were loaded on 5–12% w/v acrylamide gradient gel (1 mm). The protein concentration was determined by Bradford and electrophoresis was performed in a ProteanIIxi cell (Bio-Rad, Hercules, CA, USA) at 4 degree by supplying constant voltage of 60 for 60 min and 120 volts until the protein bands were separated. The protein gels were photographed with digital camera and examined for the further analysis.

### Statistical analysis

A completely randomized design was used with three replicates for five treatments. An individual Student’s *t* test and Tukey’s test was employed to compare the means of separate replicates by using software SAS (version 9.1, USA).

## Results

### Morphology and production of reactive oxygen species

**Figure 1:**
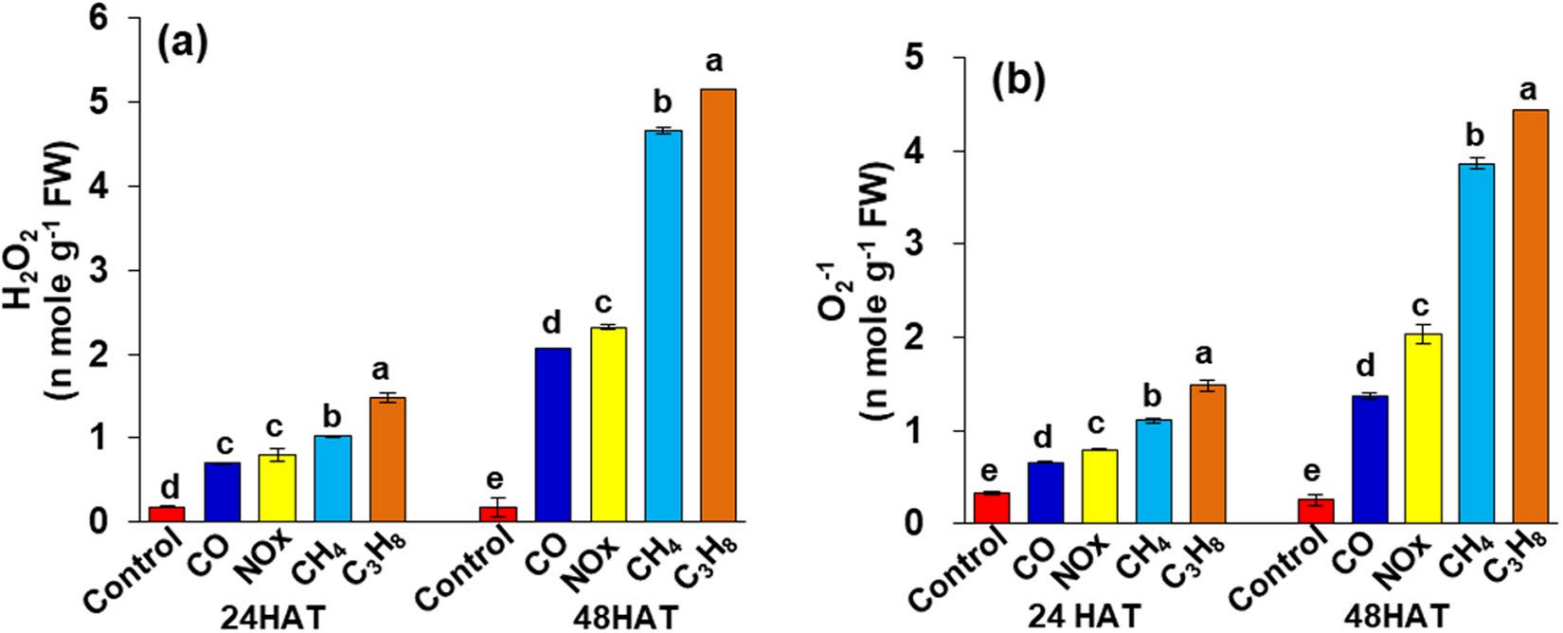
Oxidative damage in leaves indicated by (**a**) H_2_O_2_ (**b**) O_2_^−1^ determination as affected by CO, NO_x_, CH_4_ and C_3_H_8_ compared to control at 24 and 48 hours after treatment. Vertical bars indicate ± SE by means with n = 3. Means denoted by the different letter are significantly different at *p* < 0.05 according to the Tukey’s studentized range test.

The reactive oxygen species in hazardous gas-treated leaves were determined to be H_2_O_2_ and O_2_^−1^. The concentration of H_2_O_2_ in the presence of CO, NO_x_, CH_4_, and C_3_H_8_ increased significantly 24 hours after treatment (−305, −370, −488 and −723%, respectively) (Fig. 1a); however, the induction of reactive oxygen species production was more severe at 48 hours after treatment when compared to the control. Similarly, the O_2_^−1^ concentration was increased at 24 hours after treatment under CO, NO_x_, CH_4_ and C_3_H_8_ compared to control plants, whereas the concentration was severe at 48 hours after treatment and was increased by −454, −718, −144 and −1442%, respectively (Fig. 1b).

### Photosynthetic pigments and stomatal observation

**Figure 2:**
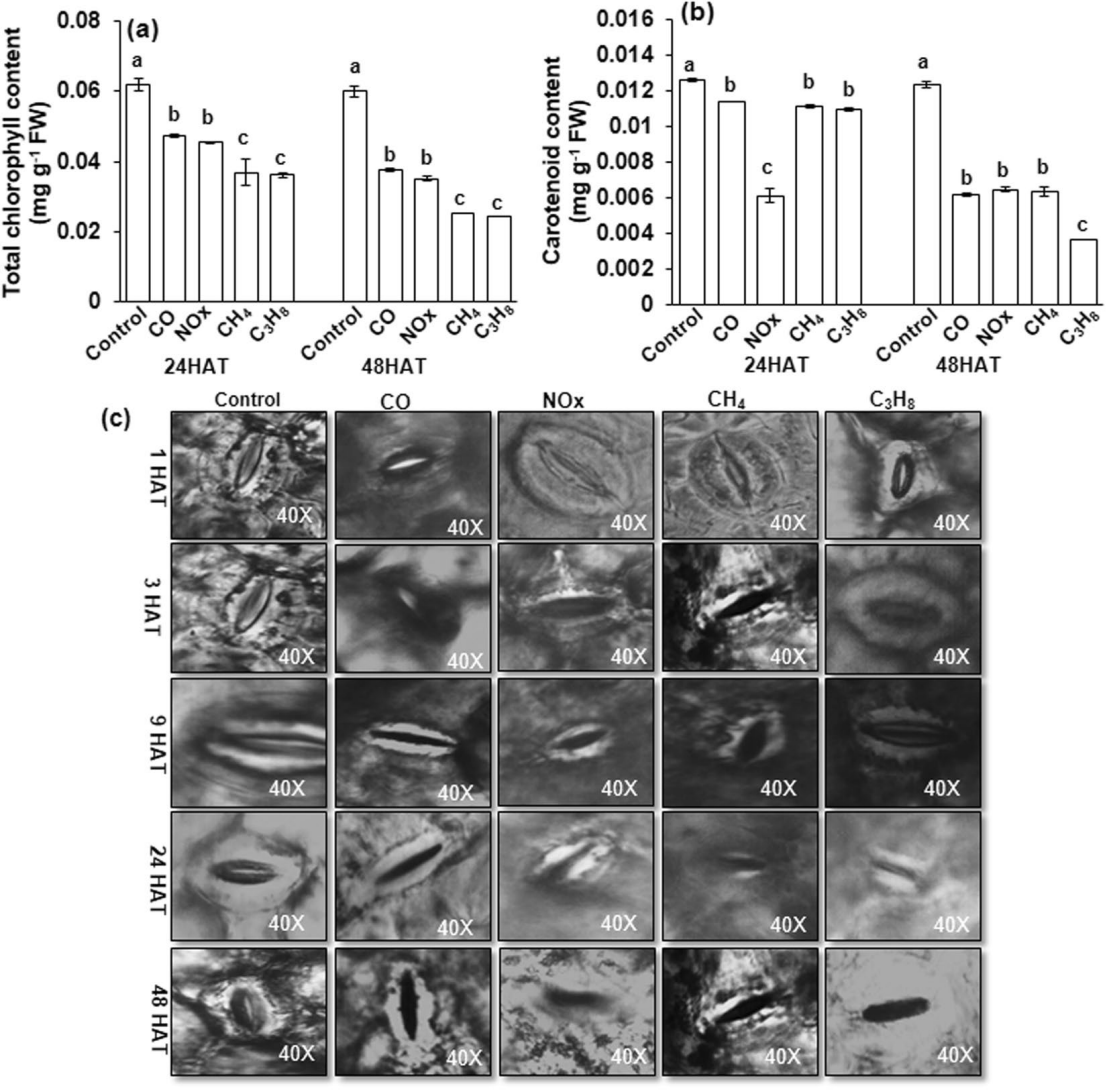
Changes in the content of photosynthetic pigments a total chlorophyll b carotenoid and c representative images of stomata as affected by CO, NO_x_, CH_4_ and C_3_H_8_ for 1–48 hours after treatment. Vertical bars indicate ± SE by means with n = 3. Means denoted by the different letter are significantly different at *p* < 0.05 according to the Tukey’s studentized range test. For stomatal observation thin layer of leaf outer covering were peeled off carefully and laid on a glass slide, covered with a cover slip and were observed under a light microscope Leica CME, Japan at 40X magnification.

The total chlorophyll and carotenoid contents were significantly decreased by CO, NO_x_, CH_4_, and C_3_H_8_ compared to control plants (Fig. 2a,b). The negative effect of these photosynthetic pigments was more prominent at 48 hours after treatment and was reduced by 50–66% of total chlorophyll content and 40–50% of carotenoid content.

Microscopic observations (40X) showed that the stomata did not show any change in behavior for the first 1 hour after CO, NO_x_, CH_4_ and C_3_H_8_ treatment (Fig. 2c). After 3 hours of treatment, the stomata closed, although the guard cells did not show any change. Conversely, 3 to 24 hours after CO, NO_x_, CH_4_ and C_3_H_8_ treatment, the stomata were completely closed, and the guard/subsidiary cells were damaged compared to the control. This damage was severely more exaggerated at 48 hours after treatment at a particular magnification chosen under a microscope.

### Photosynthetic activity and Fv/Fm ratio

**Figure 3:**
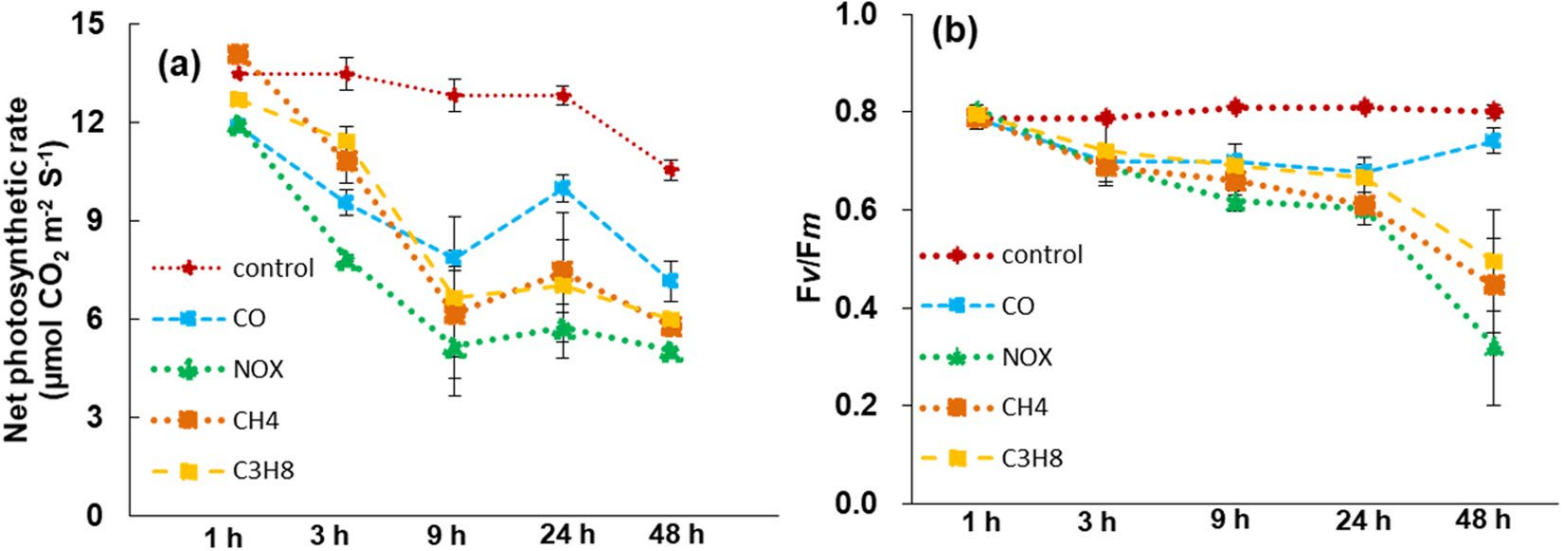
Changes in photosynthetic parameters a photosynthesis rate, b F*v*/F*m* ratio as affected by CO indicated by blue line, NO_x_ indicated by green line, CH_4_ indicated by orange line and C_3_H_8_ indicated by yellow line for 1, 3, 9, 24 and 48 hours after treatment. Lines indicate ± SE of means with n = 3.

The net photosynthetic rate did not show any changes for the first hour under CO, NO_x_, CH_4_ and C_3_H_8_ treatment; however, after 3 to 24 hours of treatment, the net photosynthetic rate decreased continuously (Fig. 3a), and the reduction in photosynthesis was observed to be highest after 48 hours of treatment compared to control plants.

Similarly, the ratio between F*v*/F*m* under CO, NO_x_, CH_4_ and C_3_H_8_ treatment did not change up to 1 hour after treatment (Fig. 3b). After 3 hours of hazardous gas treatment, the ratio of F*v*/F*m* decreased continuously, and the highest reduction was observed at 48 hours after treatment, at 12.5%, 62%, 50%, 38%, respectively, when compared to the control.

### Activity of RuBisCO and sucrose synthase

**Figure 4:**
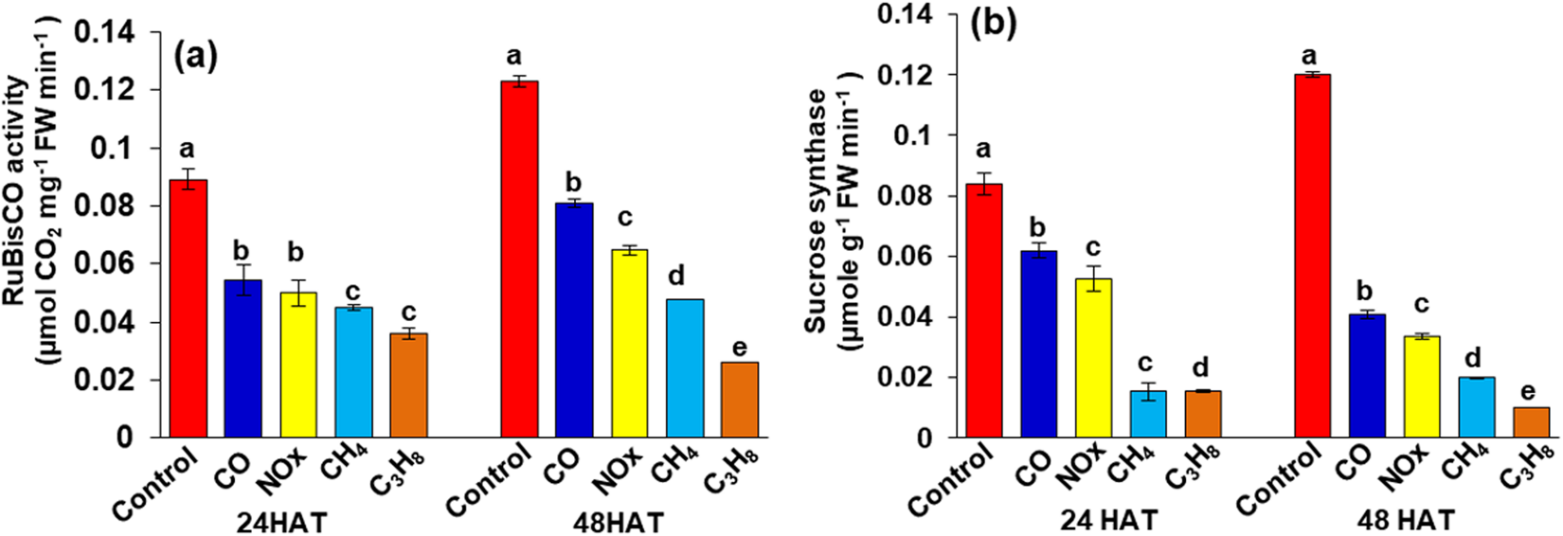
Changes in enzyme activities a RuBisCO activity b Sucrose synthase as affected by CO, NO_x_, CH_4_ and C_3_H_8_ for 24 and 48 hours after treatment. Vertical bars indicate ± SE by means with n = 3. Means denoted by the different letter are significantly different at *p* < 0.05 according to the Tukey’s studentized range test.

The response of RuBisCO and sucrose synthase activity to 24 hours of CO, NO_x_, CH_4_ and C_3_H_8_ treatment was slightly reduced (Fig. 4a,b), whereas the most severe decrease in activity was found 48 hours after treatment, at 33%, 50%, 66% and 83%, respectively, compared to control plants.

### Thylakoid multiprotein complex proteins (MCPs)

**Figure 5:**
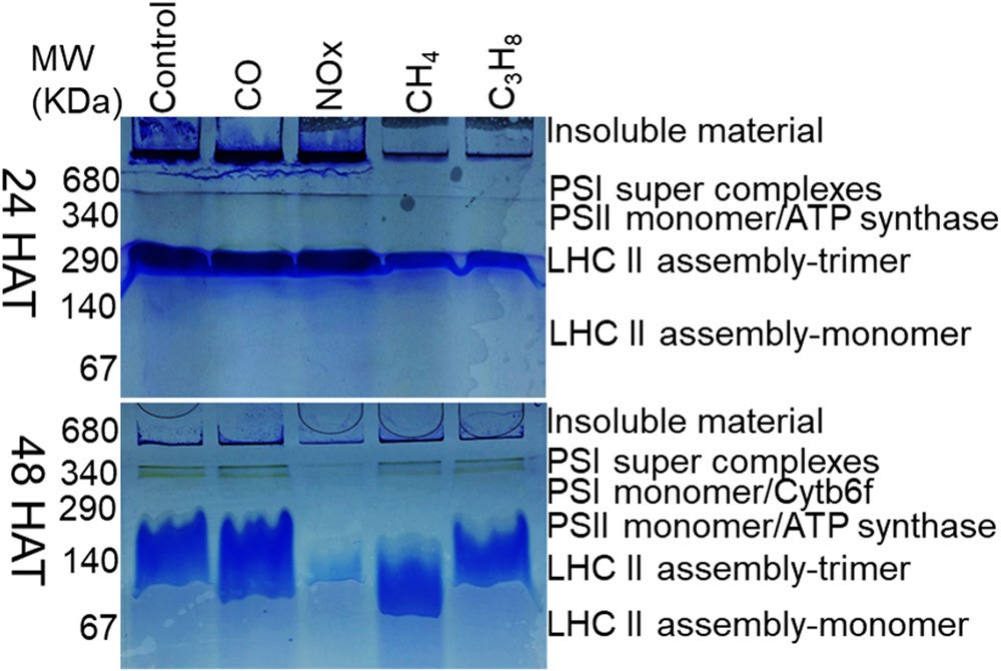
Analysis of thylakoid protein complex by BN-age as affected by CO, NO_x_, CH_4_ and C_3_H_8_ for 24 and 48 hours after treatment. Freshly thylakoid membranes from mature leaves were solubilized in 1% BDM at chlorophyll concentration of 1 µg µl^−1^, and the protein sample was separated by 7–10% gradient BN-PAGE.

First-dimensional electrophoresis run under native conditions on BN–PAGE was used to separate multiprotein complexes (MCPs) in intact form from thylakoids that were isolated from leaves affected by CO, NO_x_, CH_4_ and C_3_H_8_ (Fig. 5) at 24 and 48 hours after treatment (Supplementary Fig. S2).

Gel portions between 680–340 kDa contained a PSII–core dimer band (band 1) and PSII–core monomer super complex band (band 2). The intensity of these bands was reduced in CO, NO_x_, CH_4_ and C_3_H_8_ plants, whereas these bands were abundant in control plants at 48 hours after treatment, although the effect was not observed to be severe at 24 hours after treatment. Analogously strong variation was observed in gel portions between 340–290 kDa (bands 3–4), which contained PSII-monomer/ATP synthase and PSI monomer/cytb6f. These bands were severely reduced at 48 hours after treatment. A LHCII trimer was identified at 140 kDa (band 5). Variation in this band was not observed under hazardous gas treatment. LHC-II monomers identified at 67 kDa (band 6) (bands below 67 kDa) also remained unaffected and were expressed only at 24 hours after hazardous gas stress.

### Relationships among descriptive parameters of net photosynthesis and relative parameters

Linear correlations among descriptive parameters of net photosynthetic activity and relative pigments were assessed using the values of plants affected by C, NO, CH_3_, and C3H_8_ combined treatments along with the values of control plants (Table 1). Rubisco and total chlorophyll in leaves were closely correlated (*P* < 0.001) after 48 hours of treatment, while significant values were observed 24 hours after treatment. The correlation of carotenoid with Rubisco and total chlorophyll was significant, with *P* < 0.01. The relationships of net photosynthesis with F_v_/F_m_, Rubisco, total chlorophyll and carotenoid were highly significant after 48 hours of treatment.

**Table 1:** Linear correlations among the descriptive parameters of Net-photosynthesis, and photosynthetic parametres.

	Net photosynthesis	Fv/Fm	Rubisco	Total chlorophyll	Carotenoid
**(24 HAT)**					
Net photosynthesis	—	r = 0.554*	r = 0.385*	r = 0.562*	r = 0.098
Fv/Fm	r = 0.554*	—	r = 0.62**	r = 0.557*	r = 0.356
RuBisCO	r = 0.385	r = 0.62**	—	r = 0.842***	r = 0.140
Total chlorophyll	r = 0.526*	r = 0.557*	r = 0.842***	—	r = 0.058
Carotenoid	r = 0.098	r = 0.399	r = 0.1688	r = 0.058	—
**48 HAT**					
Net photosynthesis	—	r = 0.962***	r = 0.934***	r = 0.953***	r = 0.931***
Fv/Fm	r = 0.411	—	r = 0.395	r = 0.387	r = 0.258
RuBisCO	r = 0.934***	r = 0.395	—	r = 0.931**	r = 0.848**
Total chlorophyll	r = 0.953***	r = 0.387	r = 0.931**	—	r = 0.869**
Carotenoid	r = 0.931**	r = 0.258	r = 0.848**	r = 0.869**	—

The values measured at 24 and 48 HAT after CO, NO_x_, CH_4_ and C_3_H_8_ treatment were used for correlation analysis. The correlation coefficient (r) and significant differences are given; n = 16. *P ≤ 0.05; **P ≤ 0.01; ***P ≤ 0.001.

## Discussion

The positive effect of CO_2_ fertilization and/or a direct heating system on several horticultural crops has been reported previously^18,19–21^. However, the emission of several hazardous gases by inefficient CO_2_ fertilization/or heating systems has caused a great threat of low crop production in certain countries. In our study, we described that these hazardous gases emitted by CO_2_ supply from a direct heating system cause unavoidable by-products of aerobic metabolism, as also described in our previous studies under the influence of CO, NO_x_ and SO_2_^11^. Under normal growth conditions, ROS levels are modest, and cells experience only mild oxidative stress, whereas many stresses enhance ROS production^10,22^. The results of our study clearly showed that hazardous gases in the form of CO, NOx, CH_4_ and C_3_H_8_ generated a rapid escalation in H_2_O_2_ and O_2_^−1^ content in strawberry shoots (Fig. 1). The enhanced production of ROS can pose a threat to cells, but it is also believed that ROS serve as signaling molecules to activate the stress response and defense pathways^22,23^. Thus, the CO_2_ supply from a direct heating system or combusted gas from a hot water boiler system used in greenhouses induced ROS, which can be regarded as a cellular display of stress and as ancillary messengers involved in the stress response signal transduction pathways, causing reduced crop yield. This also led to the hypothesis that hazardous gases result in proteolytic damage and, in turn, oxidative stress^11^.

In this study, the first and foremost effect of hazardous gases (CO, NO_X_, CH_4_ and C_3_H_8_) was a reduction in photosynthetic pigment content, such as total chlorophyll and carotenoid content (Fig. 2a,b). The loss of chlorophyll and carotenoid content indicates that photodamage might have occurred in chloroplasts^24^. The loss of chlorophyll under other abiotic stresses, such as drought, salinity, and other heavy metal toxicities, has been rigorously documented in several plant species^25–29^. From previous studies, such a decrease in chlorophyll content may be attributed to a reduction in hazardous gases for the formation of precursor molecules, such as δ–aminolevulinic acid and protochlorophyllide^11,30^. An additional loss of photosynthetic pigments leads to damage in stomatal guard cells (Fig. 2c) and a reduction in stomatal density. Previous studies on stomata under abiotic stresses have also indicated an effect on the opening and closing of stomata^30–34^. In our studies, we observed that hazardous gases not only affected the opening and closing of stomata but also severely damaged guard cells/subsidiary cells. This negative effect on guard cells and density in our results indicates that leaves might have been defoliated during the growth conditions due to high temperatures under hazardous gas treatments^35–37^. The closure of stomata due to hazardous gases might be due to the synthesis and mobilization of abscisic acid (ABA) due to key physiological actions in plants, which result in the closure of stomata^38–40^. Based on the current knowledge of stomatal behavior, it is quite obvious that gaseous exchange was likely to be affected by the reduction in stomatal conductivity and the transpiration rate^41^, which leads to the reduction in net photosynthesis and the ratio of F*v*/F*m* (Fig. 3). The reduction in net photosynthesis and the ratio between F*v*/F*m* has been observed under various other abiotic stresses, such as in green algae under salinity^42^, tomato^43^, oilseed rape under Fe deficiency^44^ and strawberry under CO, NO_X_ and SO_2_ stresses^11^. The reduction in net photosynthesis and F*v*/F*m* might be due to inactivation of photosynthesis reaction centers, which obtain a preliminary quantity of light energy that cannot exploit efficiently due to the presence of oxidative stress. This ineffective energy exploitation^45^ leads to an extreme increase in the dissipated energy and therefore decreases photosynthesis. The decrease in photosynthetic pigments and stomatal behavior under hazardous gases resulted in the reduction in the activity of important enzymes such as RuBisCO and sucrose synthase. The degradation of RuBisCO and sucrose may imply that other housekeeping enzymes may lead to a decline in multiprotein complexes, which are important for light and dark reactions during photosynthesis, as also shown by our results (Fig. 5).

The study of biomacromolecules (multiprotein thylakoid membrane proteins), which are essential proteins for photosystems I and II, under hazardous gases has not been conducted until now. To determine whether the multiprotein complexes of chloroplasts were affected, 1^st^-dimensional BN-PAGE of chloroplast/thylakoid complexes in strawberry leaves under hazardous gas treatments was performed (Fig. 5). We studied thylakoid proteins after 48 hours of hazardous gas treatment, and strong reductions in PSII–core dimer, PSII–core monomer/ATP synthase, PSI monomer/cytb6f and LHCII trimer complex were observed. The consequent high availability of hazardous gases (CO, NO_x_, CH_4_ and C_3_H_8_) may limit the activity of (4Fe-4S) and (2Fe-2S) clusters, which are associated with chloroplast membranes, especially in the leaves of strawberry, one of the highest demanding plants for fruits throughout the year. Thus, it can be concluded that chlorosis is possibly due to the proteolytic loss of photosystems, the cytb6/f complex^46^ and the light–harvesting chlorophylls and carotenoids. It has been generally established that leaf chlorosis is accompanied by a reduction in the number of granal and stromal lamellae per chloroplast and is due to a lower transpiration rate and stomatal conductivity^47^.

The present data clearly showed that hazardous gases crucially produce ROS and drastically limit photosynthesis by disruption of the chloroplast membrane and by the accompanied proteolytic losses of photosystems I and II in the presence of CO, NO_x_, CH_4_, and C_3_H_8_. In addition, the decrease in the activity of RuBisCO and sucrose synthase, net photosynthesis, the F*v*/F*m* ratio and stomatal behavior in response to hazardous gases was paralleled with the responses of thylakoid protein complexes, and the structure of the stem was observed to determine the influence of hazardous gases on cortical cells. CO_2_ fertilization from a direct heating system greatly emits hazardous gases, which lead to a reduction in crop yield. From the present study, we conclude that the older CO_2_ boilers/heating systems installed in greenhouses need improved efficiency, and this can be done by using sensors to analyze the emission of hazardous and hydrocarbons. Therefore, the present results can be used to prepare manuals for protecting horticulture to improve agricultural products, particularly for commercial purposes. Moreover, the results of chloroplast protein analysis revealed an initial step towards the molecular pathways of the photosynthetic mechanisms of strawberry grown inside a closed environmental system. Overall, the current results revealed that major steps must be taken to introduce resourceful CO_2_ fertilization from a direct heating system to reduce the risks to crop yield globally, particularly in those countries where the risk of these hazardous gases is still causing considerable crop productivity loss. More studies on molecular mechanisms, such as chloroplast proteomics, will be useful to identify stress-responsive proteins affected by hazardous gases, and indeed, those results can also be used to identify resistant and sensitive cultivars. In addition, plant breeders can select specific cultivars for particular environmental systems.

## Electronic supplementary material

Supplementary information
