# Supplementary material for: Hazardous gases (CO, NOx, CH4 and C3H8) released from CO2 fertilizer unit lead to oxidative damage and degrades photosynthesis in strawberry plants
Source: Sci Rep. 2018 Aug 16;8:12291. doi: 10.1038/s41598-018-30838-3 (PMC6095870; doi:10.1038/s41598-018-30838-3)
Supplement: Supplementary file 1 — Supplementary information [file 41598_2018_30838_MOESM1_ESM.docx]

**Hazardous gases (CO, NOx, CH_4_ and C_3_H_8_) released from CO_2_ fertilizer unit lead to oxidative damage and degrades photosynthesis in strawberry plants**

**Sowbiya Muneer^1,2,3^, and Jeong Hyun Lee^1†^**

^1^Department of Horticulture, College of Agricultural life sciences, Chonnam National University, 300 Young Bong–Dong Buk–Gu, Gwangju, 500–757, Korea.

^2^Centre for Agricultural Innovations and Adavnced Learning [VAIAL], Vellore Institute of Technology, Tamil Nadu, Vellore-632014, India.

^3^School of Bioscience and Biotechnology, Vellore Institute of Technology, Tamil-Nadu, Vellore-632014, India.

^†^Corresponding author: [leetag@chonnam.ac.kr](mailto:leetag@chonnam.ac.kr)

Alternate corresponding author email id: [sowbiya.muneer@vit.ac.in](mailto:sowbiya.muneer@vit.ac.in)


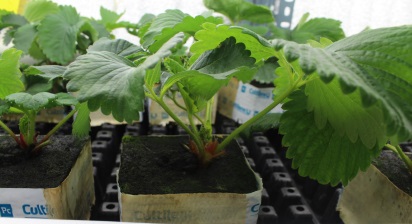


**Strawberry plants propagated on rock wool cubes under fluorescent light in hydroponic medium for 3 weeks**


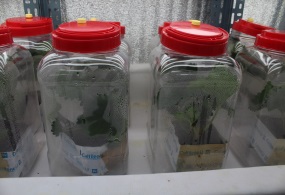

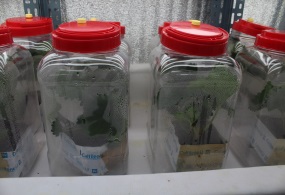

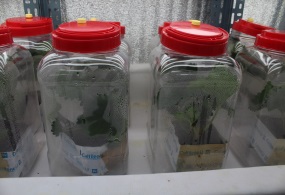

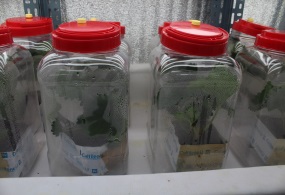


**Control**

**CO**

**NOx**

**CH_3_**

Injecting different concentration of gases

**Harvest 1-48 hours after treatment**

step1

Step 2

Step 3

Step 4

Step 5

**Experimental analysis**

After 6 weeks plants transferred to air-tight bottles (3 liters)


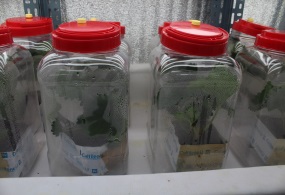


**C_3_H_8_**

**Fig S1:** Diagrammatic demonstration of experimental methodology (Note: Drawn by the authors, all pictures used in the figure is photographed by authors)


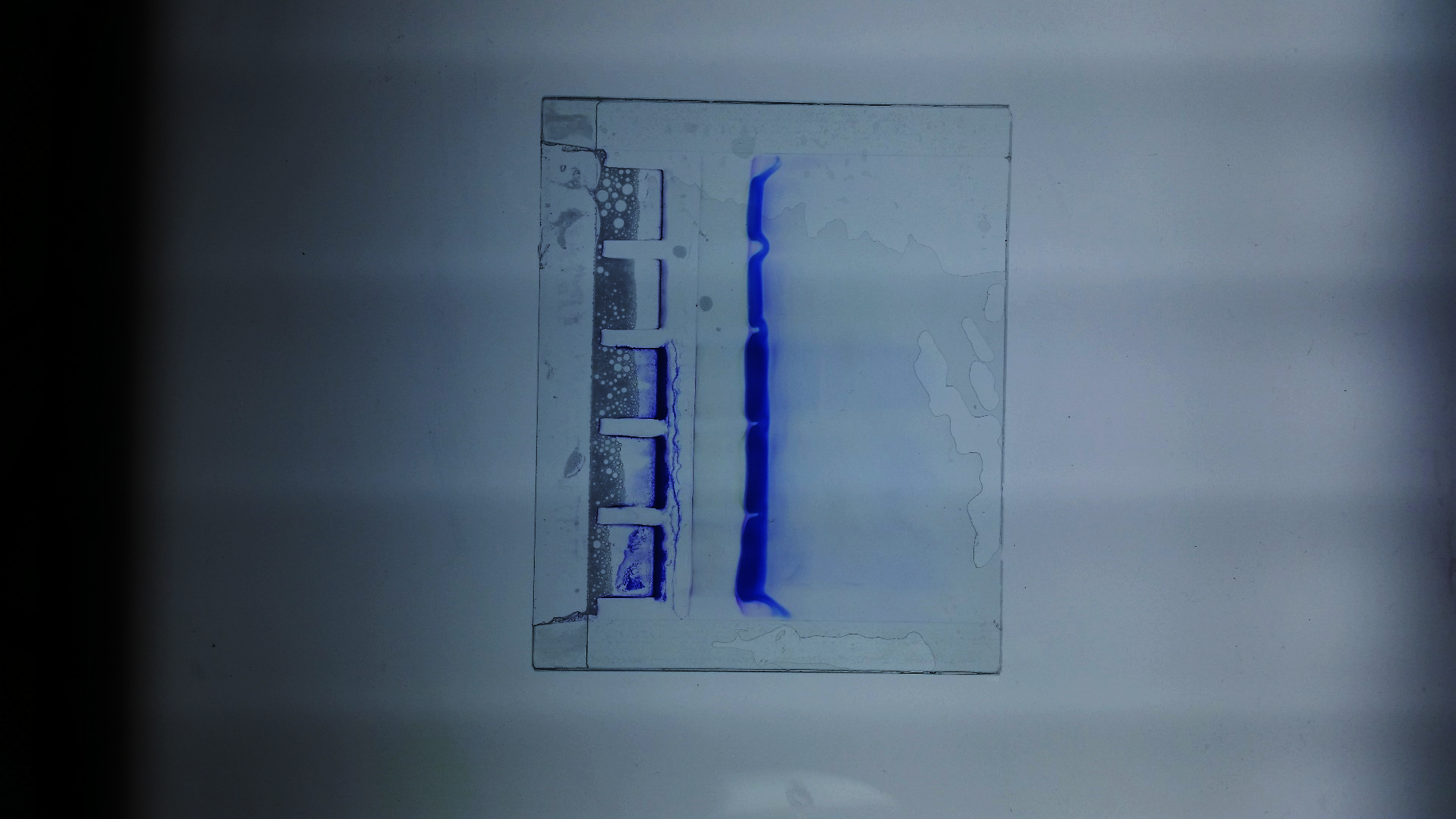

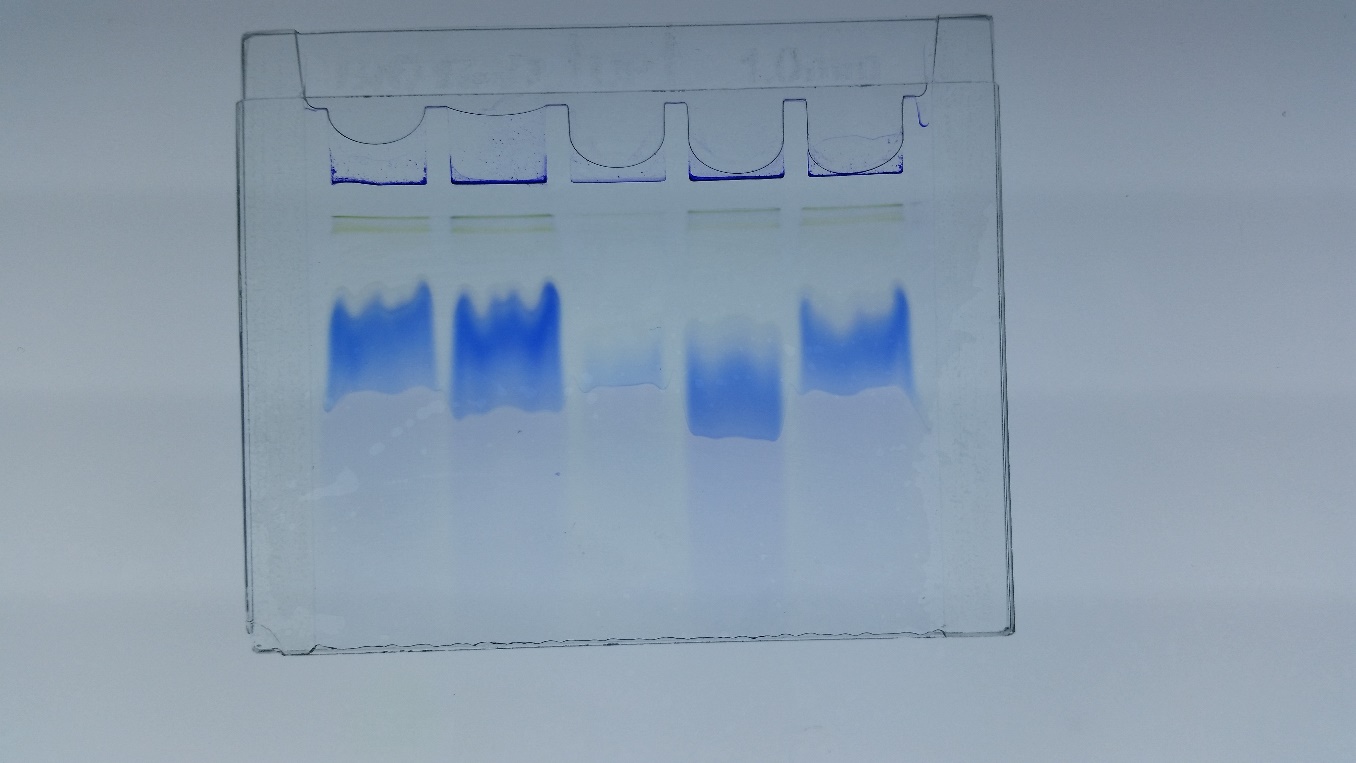


Control

CO

NOx

CH_4_

C_3_H_8_

Original images (uncropped)

48 HAT

24 HAT

**Figure S2**: Uncropped BN-PAGE gels as affected by CO, NO_x_, CH_4_ and C_3_H_8_ for 24 and 48 hours after treatment. Freshly thylakoid membranes from mature leaves were solubilized in 1% BDM at chlorophyll concentration of 1µg µl^–1^, and the protein sample was separated by 7–10% gradient BN–PAGE.
